# Low Heterozygosity and Historical Bottleneck Effect Depicted From the Genome Assembly of the Indus River Dolphin ( *Platanista minor* )

**DOI:** 10.1002/ece3.71462

**Published:** 2025-05-25

**Authors:** Aamir Ibrahim, Simin Chai, Cuijuan Zhong, Kang Jieqiong, Ahsaan Ali, Sajjad Hussain, Hassan Ali, Tanveer Hussain, Umer Waqas, Guang Yang

**Affiliations:** ^1^ Jiangsu Key Laboratory for the Biodiversity Conservation and Sustainable Utilization in the Middle and Lower Reaches of the Yangtze River Basin, College of Life Sciences Nanjing Normal University Nanjing China; ^2^ Southern Marine Science and Engineering Guangdong Laboratory (Guangzhou) Guangzhou Guangdong China; ^3^ Wildlife and Parks Department Lahore Punjab Pakistan; ^4^ Department of Biological Sciences Virtual University of Pakistan Islamabad Pakistan; ^5^ Virtual University of Pakistan Lahore Pakistan

**Keywords:** demography, endangered, freshwater adaptation, homozygosity, threats

## Abstract

The Indus River dolphin ( *Platanista minor* ) is a highly endangered freshwater dolphin endemic to the Indus River system of the Indian subcontinent. We reported a *de novo* assembly and characterization of the draft genome of the Indus River dolphin by using Illumina short‐read sequencing technology. Based on this, for the first time, we conducted the comparative genomics study and identified a selection of genes and gene families that have undergone significant positive selection and expansion or contraction, indicating potential molecular mechanisms associated with freshwater adaptation, such as specialized skin features and their derivatives (e.g., hair loss) and immune adaptations. Additionally, this study estimated that the Indus River dolphin diverged nearly 31.2 million years ago from the most recent common ancestor of Delphinidae and Lipotidae, placing it in a more basal position to other freshwater dolphins (e.g., the baiji *Lipotes vexillifer* ). It was suggested that the combined effects of the natural historical bottleneck effect around 40–20 kiloyears ago and anthropogenic activities were the driving factors of inbreeding for this species with very low heterozygosity (0.0218%).

## Graphical abstract

The Indus River dolphin ( *Platanista minor* ) is a highly endangered freshwater dolphin endemic to the Indus River system of the Indian subcontinent. We reported a relatively high‐quality de novo genome assembly of it. We revealed the low heterozygosity as well as the molecular mechanisms underlying its adaptation and evolution.

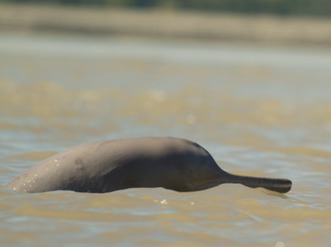

## 1 Introduction

The riverine *Platanista* lineage is an exclusively extant member of the extinct Platanistidae (de Muizon 1988, Viglino et al. 2021), which might have escaped extinction due to an earlier transition from a marine to a freshwater environment (Hamilton et al. 2001). The phylogeny of the extant *Platanista* spp. has been a subject of longstanding debate. Now, it is accepted that *Platanista* is an early‐diverging lineage, occupying the most basal phylogenetic position among river dolphins; and the observed convergent features among riverine taxa are plesiomorphic characters (Árnason and Gullberg 1996; Cassens et al. 2000; Yan et al. 2005; Werth 2006; McGowen et al. 2009; Steeman et al. 2009; McCurry et al. 2017).

**FIGURE 1:**
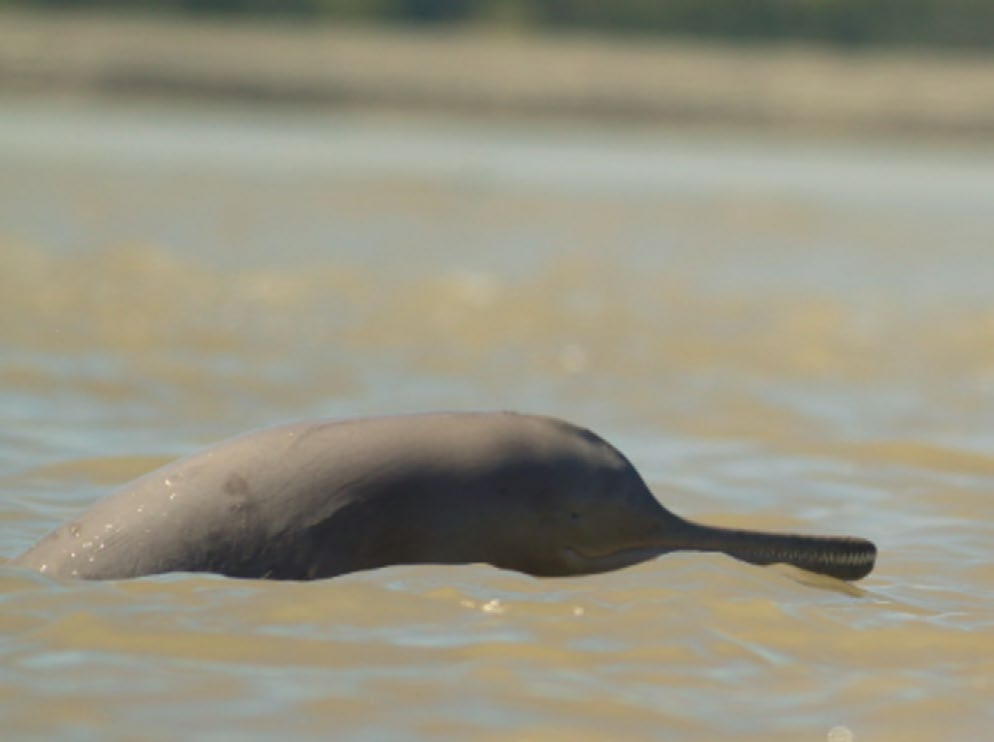
The Indus river dolphin in natural habitat, Indus River, Pakistan (Picture Credit: Dr. Aamir Ibrahim).

It has been suggested that *Platanista* evolved during the late Oligocene, with high diversity recorded in the early Miocene and markedly decreased by the middle‐late Miocene (de Muizon 1988; Fordyce and de Muizon 2001). During the late Neogene epoch, *Platanista* underwent a transition to the freshwater environment of the Ganges River system before subsequently migrating to the Indus River system (Hamilton et al. 2001). The presence of distinct geographical distribution and reproductive isolation has resulted in the recognition of two separate species: the Ganges River dolphin ( *Platanista gangetica* ) endemic to the Ganges River system, and the Indus River dolphin ( *P. minor* ) (Figure 1) endemic to the Indus River system; both are endangered species (Braulik et al. 2015; Braulik et al. 2021). Specifically, the drastic negative anthropogenic impacts such as hunting and development of the Indus Basin irrigation system (after 1885) drove the Indus River dolphin to the brink of extinction (Anderson 1878; Braulik et al. 2015). It is gratifying to note that, since the 1970s, conservation efforts have enabled its resurgence, with current estimates exceeding 2000 individuals throughout their distribution range (WWF‐Pakistan 2017).

Although former studies focused on the mitochondrial genome of one subpopulation (Sindh Province, Pakistan) and reported high homozygosity (Braulik et al. 2015), investigations into other subpopulations are lacking. Fragmented, smaller, and isolated populations are prone to a higher risk of extinction due to increased inbreeding, resulting in reduced genetic diversity and diminished adaptive potential to sustained viability (Lande 1993; Reed and Frankham 2003; Willi et al. 2006; Melbourne and Hastings 2008). Importantly, evolutionary and demographic histories are essential for the development of an efficient conservation strategy (Nigenda‐Morales et al. 2023), although they are still undisclosed for the Indus River dolphin.

Over the last four decades, genetics has played a vital role in promoting the conservation of endangered species. The genetics data has assessed the genetic variation at individual and population levels; the genetics has also provided deep insights in diverse areas of conservation biology including species identification, hybridization, kinship, evolutionary history, effective population size (*N*_e_), population substructure, population connectivity, and inbreeding (Hedrick and Miller 1992; von Der Heyden et al. 2014; Haig et al. 2016). To address these issues, whole genome sequencing serves as a valuable tool for uncovering the unknown biological characteristics of species. A range of cetaceans have been examined through genomics research at both the individual and population levels. For example, through whole genome sequencing and comparative genomics analyses of the Baiji ( *Lipotes vexillifer* ), the flagship species of the Yangtze River in China, insights into molecular adaptation, decreased heterozygosity, and a historical bottleneck have been gained (Zhou et al. 2013). Other examples include the sperm whale ( *Physeter macrocephalus* ) (Warren et al. 2017), the bottlenose dolphin ( *Tursiops truncatus* ) (Foote et al. 2015), the Yangtze finless porpoise ( *Neophocaena asiaeorientalis* ) (Zhou et al. 2018), and the pygmy right whale ( *Caperea marginata* ) (Wolf et al. 2023), and so forth. However, whole genome sequencing and annotation are rare for river dolphins. Especially, only one available draft genome is accessible for the Indus River dolphin from an unknown subpopulation (https://www.ncbi.nlm.nih.gov/datasets/genome/GCA_004363435.1/).

In this study, we reported the newly assembled draft genome and annotation of the Indus River dolphin, which was sequenced at approximately 100× sequencing coverage. Comparative genomic analyses yielded insights into molecular adaptive characteristics in a riverine habitat and shed light on its enigmatic demographic population history. This study will contribute to the genetic conservation efforts for this highly endangered riverine dolphin.

## 2 Material and Methods

### 2.1 Sampling and Sequencing

The source of the current genomic study was conducted based on the single adult female Indus River dolphin. This individual was collected on (January‐14‐2022) from the subpopulation between the Taunsa–Guddu barrage (28.921437^o^N, 70.541958°E) in the Punjab River section (Pakistan). As this species has endangered status and rarely has availability of fresh samples for genetic study, we preserved it at −20°C until performing DNA extraction and genome sequencing on June 15, 2023. In our collection, only one adult female sample adequately passed the quality to avail only illumina short‐read sequencing technology. Deep skeletal muscle tissues from the abdomen region were selected to process for genomic study, and DNA extraction was performed using the phenol/chloroform DNA extraction method. Paired‐end sequencing libraries, with an insert size of ~350 bp, were constructed and sequenced by the independent sequencing platform zero PCR DNBSEQ from BGI (Quail et al. 2012).

### 2.2 Genome Assembly and Annotation

To assemble and annotate the newly sequenced Indus River dolphin genome, SOAPnuke (v1.6.5) was used to filter data and get clean reads, including (1) removing reads with 25% low‐quality bases (quality scores ≤ 7), (2) removing reads with N bases more than 1%, (3) discarding reads with adapter contamination and/or polymerase chain reaction duplicates (Chen et al. 2018). We used the Jellyfish (v2.1.4) (Luo et al. 2012) software based on a *K*‐mer distribution and GenomeScope2 (v2.0) (Vurture et al. 2017) to estimate the genome size with high‐quality reads > Q20. The Platanus (v1.2.4) (Kajitani et al. 2014) was used for *de novo* genome assembly and scaffold construction using the default parameters. To evaluate the quality of the present genome assembly, we assessed its integrity using Benchmarking Universal Single‐Copy Orthologs (BUSCO v5.2.2) with a database of mammals (mammalia_odb10) (Simão et al. 2015).

We annotated the repeat sequences in the present Indus River dolphin genome assembly using both *de novo* predictions and homology‐based searching in the known repeat database. RepeatMasker (v4.1.1) and RepeatProteinMasker (v4.1.1) RepBase23.06 (https://www.girinst.org/repbase/) were applied to identify repetitive elements at the DNA and protein levels, respectively. RepeatModeler (v2.0.3) was performed for *de novo* prediction, and Tandem Repeats Finder (v4.09.1) (Benson 1999) was applied to annotate tandem repeats.

Protein‐coding genes were annotated using homology‐based and *de novo* prediction. We annotated protein‐coding genes using MAKER (v3.01.03) (Cantarel et al. 2008) based on three independent approaches, including homology‐based, ab initio, and RNA sequencing‐assisted prediction. The GeMoMa (v1.9) (Keilwagen et al. 2016) software was used to align homologous peptides from cattle *Bos taurus* (GCF_002263795.3), human *Homo sapiens* (GCF_000001405.40), baiji (GCF_000442215.2), mouse *Mus musculus* (GCF_000001635.27), sperm whale (GCF_002837175.3), and bottlenose dolphin *Tursiops truncatus* (GCF_011762595.1) to the assembly and infer the gene structure information. For RNA seq‐based gene prediction, mRNA‐seq reads from the closely related bottlenose dolphin (DRR513290, DRR513294, DRR513301, and DRR513302) were aligned to the assembly and then assembled to predict open reading frames using Stringtie (v2.2.1) (Pertea et al. 2015). Augustus (Stanke et al. 2008), GeneMark‐ETP (Brůna et al. 2020), and SNAP (Korf 2004) were then used for ab initio gene prediction on the training set from GeMoMa.

Gene function information was assigned by comparing them with public databases including Swiss‐Prot, Nr, KEGG, and Gene Ontology. To assess the non‐coding rRNA, tRNA, snRNA, snoRNA, and microRNA, we used INFERNAL (v1.1.4) to search the Rfam database (Nawrocki et al. 2009).

Through the aforementioned methods of homology‐based gene structure annotation and gene function annotation, we proceed to annotate the protein‐coding genes of the publicly available draft genome assembly GCA_004363435.1.

### 2.3 Phylogeny, Demographic History and Heterozygosity Level

The phylogenetic tree was reconstructed based on the coding sequences of 8806 single‐copy orthologous genes identified by Orthofinder (v2.5.3) (Emms and Kelly 2015) with default parameters from the Indus River dolphin, the sperm whale, the blue whale *Balaenoptera musculus* , the Baiji, the bottlenose dolphin, the cattle, the mouse, with the human as an outgroup. We used MAFFT (v7.520) (Katoh and Standley 2013) for multiple sequence alignment and used RAxML (v8.2.12) (Stamatakis 2014) to reconstruct the phylogenetic tree. The species divergence time was estimated using MCMCTREE in PAML (v4.9) (Yang 2007). Three calibration points were retrieved from Timetree (http://www.timetree.org/), including human ~ the mouse (81.3–91.0 MYA), the blue whale ~ the bottlenose dolphin (32.3–35.2 MYA), the sperm whale ~ the bottlenose dolphin (32.1–34.3 MYA). MCMCTREE with parameters “clock = 3, model = 0, alpha = 0, ncatG = 4, BDparas = 1 1 0, finetune = 0.05 0.1 0.12 0.1 0.3, burnin = 20000, nsample = 100000” was used to infer divergence time.

For the demographic history, we combined both the already available genome and newly generated genome of the Indus River dolphin; the data was inferred with the PSMC model (Li and Durbin 2009). We set *N* = 25, *t* = 15, *r* = 5, and *p* = “4 + 25*2 + 4 + 6” for PSMC analysis with 100 bootstrap replicates. The value of N, set at 25, signifies that the maximum number of iterations is 25; with the assumption of 15 years per generation, the notation *t* = 15 represents the conversion of a generation into years; *r* is the ratio of initial theta to rho, set at 5 and near the default setting; *p* signifies the pattern of parameters and was set as default. GATK was used for the single nucleotide polymorphisms (SNPs) calling and filtering (Poplin et al. 2017). The heterozygosity level was calculated as the ratio of the number of heterozygous SNPs divided by the number of totally filtered SNPs.

### 2.4 Comparative Genomics and Evolutionary Analyses

We employed PAML (v4.9) (Yang 2007) to detect positively selected genes (PSGs) using the branch‐site model. This model aims to detect positive selection that affects only a few sites on the prespecified foreground branch(es), the Indus River dolphin in the present study. For the background branch, there are two classes of sites, the conserved sites with 0 < ω_0_ < 1 and the neutral sites with ω_1_ = 1. For the foreground branch, some of those sites become under positive selection with ω_2_ > 1. Positive selection or the presence of sites with ω_2_ > 1 is tested by comparing this model with a null model in which ω_2_ = 1 is fixed, using a 50:50 mixture of 0 and *χ* ^2^ as the null distribution. The BEB (Bayes empirical Bayes) method was used to identify codon sites potentially under positive selection on the foreground branches. While genes with the false discovery rate (FDR) of *χ* ^2^ less than 0.05 were identified as positive selection genes.

Gene family evolution was determined using CAFÉ (v4.2.1) (Mendes et al. 2020). The program uses a birth and death process to model gene gain and loss over a phylogeny. For each family in the data file, CAFÉ computes a probability (*p*‐value) of observing the data given the average rate of gain and loss of genes. Expansions and contractions of gene families with *p*‐values less than 0.05 were significant.

Enrichment of KEGG (https://www.genome.jp/kegg/) and GO (https://geneontology.org/docs/go‐enrichment‐analysis/) terms for the gene families which were significantly expanded or contracted in the Indus River dolphin was performed using clusterProfiler v4.0 (Wu et al. 2021). KEGG and GO categories with p‐adjust less than 0.05 were significant.

## 3 Results

After filtering low‐quality data and duplicate reads, we finally obtained 265.31 Gb from the Illumina platform for subsequent analyses (Table S1). An estimated genome size of 3.35 Gb was deduced from the detection of a *K*‐mer (*K*‐21) peak at 40× (Figure S1). We obtained the final draft genome assembly of a total length of 2.74 Gb and the GC‐content of 41.9%, similar to the previously reported assembly of the Indus River dolphin (PlaMin_v1_BIUU, GCA_0044363435.1). The present genome assembly exhibits contig and scaffold N50 values of 10.7 kb and 40.6 kb, respectively, which are comparable in quality to the assembly of the Indus River dolphin published previously (Table 1 and Table S2). The BUSCO analysis showed that 69.8% (single‐copied gene: 69%, duplicated gene: 0.8%) of the mammalia_odb10 gene set was identified as complete, 4.52% of genes were fragmented, and 25.7% of genes were missing in the assembled genome (Table 2 and Table S3). Although not the best BUSCO assessment for cetaceans or river dolphins, this is the first published genome annotation and genome completeness evaluation of the Indus River dolphin (Table 3).

**TABLE 1:** Summary statistics of the current draft genome assembly and assemblies' comparison of the Indus River dolphin.

Draft assembly	The present draft assembly	PlaMin_v1_BIUU
Genome size	2.74 Gb	2.67 Gb
Assembly level	Scaffold	Scaffold
Number of scaffolds	3,275,841	1,098,790
Scaffold N50	40.6 kb	23.9 kb
Number of contigs	3,511,952	1,110,492
Contig N50	10.7 kb	20.9 kb
Genome coverage	40×	28×
GC percent	41.9%	41.5%

**TABLE 2:** BUSCO evaluation of the present Indus River dolphin genome assembly.

BUSCO statistics	Genes	Percentage
Completeness	6440	69.8%
Single‐copied	6367	69.0%
Duplicated	73	0.8%
Fragmented	414	4.5%
Missing	2372	25.7%

**TABLE 3:** Comparison of BUSCO evaluations (mammalia_odb) in cetaceans.

Species	Data source	BUSCO Evaluation	Composed of repetitive sequence	Protein‐coding genes
Indus River dolphin	This study	C: 69.8%	35.64%	25,591
Baiji	GCF_003031525	C: 94.6%	43.20%	22,168
Sperm whale	GCA_000472045	C: 94.7%	40.40%	18,686
Bowhead whale	bowhead‐whale.org	C: 90.0%	41.00%	22,677
Indo‐Pacific humpback dolphin	GCA_007760645	C: 94.3%	37.41%	24,640

A total of 35.64% of the Indus River dolphin genome is composed of repetitive sequences, with the highest value recorded for long interspersed nuclear elements (LINEs, 19.75%), followed by short interspersed nuclear elements (SINEs, 6.45%), long terminal repeats (LTRs, 4.50%), DNA transposons (2.37%), and the lowest value for simple repeats (0.89%) (Figure S2 and Table S4). In the non‐coding RNA annotation, a total of 474 miRNAs, 1063 tRNAs, 180 rRNAs, and 1672 snRNAs were identified (Table S5). Our predictions indicated the presence of 25,591 protein‐coding genes, with an average gene length of 9640.82 bp. The average lengths of exons and introns were determined to be 174.86 and 1750.31 bp, respectively (Table S6).

**FIGURE 2:**
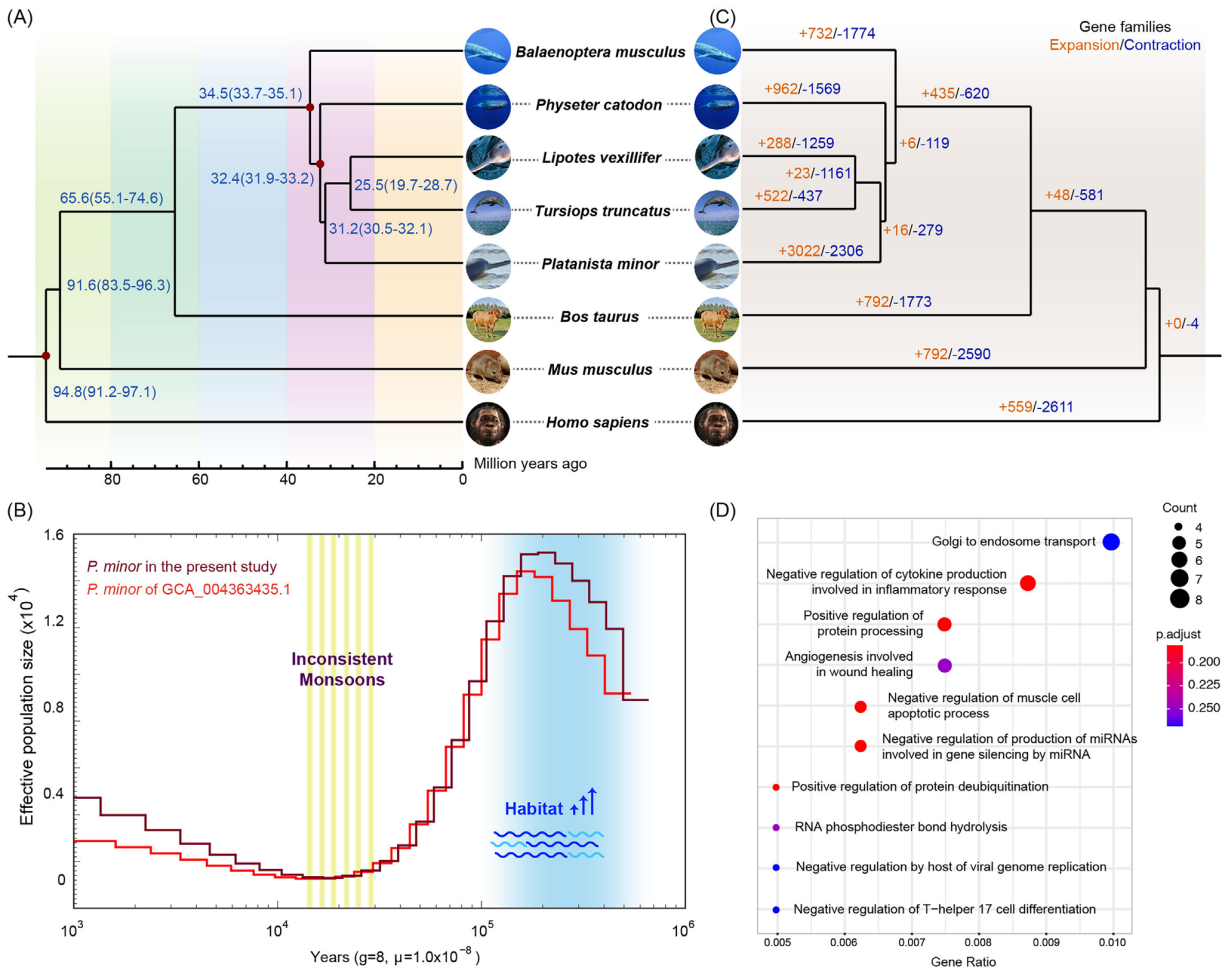
The divergence, historical population demography, and genome evolution of the Indus River dolphin. (A) A phylogram showing the phylogeny of the Indus River dolphin and other representative mammals, using the human as an outgroup. (B) Pairwise sequential Markovian coalescent result displays the historical demography. (C) Gene family evolution. Gains (in orange) and losses (in blue) of gene families are denoted along nodes and branches. (D) Gene Ontology enrichment of the positively selected genes.

Further, the phylogeny inferred from concatenated alignments of the single‐copy orthologues showed that the Indus River dolphin is in the most basal position among the riverine dolphins examined in our dataset (Figure 2A and Figure S3). Additionally, we estimated that the Indus River dolphin and the sperm whale diverged around 32.4 million years ago (MYA), while the Indus River dolphin and the common ancestor of Lipotidae and Delphinidae diverged ~31.2 MYA (Figure 2A).

GATK estimated a total of 977,685 SNPs, and after filtering, we obtained 597,874 SNP across the current genome assembly (2,746,718,295 bp). As a result, a low heterozygosity level of 0.0218% was detected. Utilizing genomic data from individuals from the present study and publicly available assemblies, the PSMC method was employed to infer changes in the effective population size (*N*_*e*_) of the Indus River dolphin. The demographic inference showed a population peak at around 0.3–0.2 MYA after a persistent increase (Figure 2B).

Based on the present assembly and genome annotation, we identified 933 expanded gene families (4238 genes) and 53 contracted gene families (46 genes) specifically in the Indus River dolphin (Figure 2C; Table S7; Figure S4). Further functional enrichment analyses showed the expanded gene families were significantly enriched in GO and KEGG terms related to immunity, nervous system, cell growth and death, translation, oxidative stress, and glycolipid metabolism. Genes within the contracted gene families were significantly enriched in keratinization, adaptive immune response, and olfactory system (Tables S8, S9, and S10; Figures S5 and S6). Moreover, we identified 878 positively selected genes (*p* < 0.05) in the Indus River dolphin (Table S11). These positively selected genes were functionally enriched in wound healing, muscle cell apoptotic process, inflammatory response, and immunity (Figure 2D; Tables S12 and S13).

## 4 Discussion

High‐quality genomics data from unstudied species can provide answers to various biological questions in conservation biology, including population demographic history, *N*_*e*_ estimation, genealogical tracing of mutations, genetics, and local adaptations (Nagasaki et al. 2015). In the present study, we showed a newly sequenced and annotated draft genome of the single adult female Indus River dolphin. Our assembly metrics are comparable to the previously reported one, which was generated using next‐generation sequencing technology similarly (Table 1). However, due to limitations in sequencing platform and technology, the continuity and completeness of our genome are not as high as the genome of the baiji, a fellow freshwater dolphin, nor do they exceed the quality of other cetaceans (Table 2). This could be addressed in the future by integrating more advanced sequencing strategies. Nevertheless, based on the current genomic information, we can to some extent comprehend the evolutionary history, genomic features, and adaptation mechanisms of the Indus River dolphin.

The genomic characteristics of the Indus River dolphin are, on the whole, similar to those of other cetaceans, showing a lower proportion of repetitive sequences and a higher proportion of protein‐coding genes compared to baiji, and a closer resemblance to the Indo‐Pacific humpback dolphin (Table 3) (Zhou et al. 2013; Keane et al. 2015; Warren et al. 2017; Ming et al. 2019).

The phylogenetic placement of *Platanista* has been the subject of ongoing debate and controversy. The distinctive morphological and skeletal characteristics of this lineage establish *Platanista* as an early‐diverging lineage, positioning it at the most basal level among river dolphins (Árnason and Gullberg 1996; Cassens et al. 2000; McHenry et al. 2006; Werth 2006; McGowen et al. 2009; Steeman et al. 2009; Xiong et al. 2009; McCurry et al. 2017). Importantly, this study supported the placement that river dolphins are not a monophyletic group and that *Platanista* has a distant relationship with another representative riverine dolphin lineage, that is, Lipotidae. This observation highlighted the independent origination and adaptation of river dolphin lineages as well as the convergent evolution. Our phylogeny and divergence times overall concorded with tree topologies and estimates in previous studies based on the fossil record and a small set of molecular markers from the mitochondrial and nuclear genes (Árnason and Gullberg 1996; Hamilton et al. 2001; Nikaido et al. 2001; McGowen et al. 2009; Xiong et al. 2009). The fossil records of extinct Platanistidae species from the late Oligocene supported the divergence time of the Indus River dolphin estimated in the present study (Braulik et al. 2015; Braulik et al. 2021).

It has been suggested that the *Platanista* lineage entered the freshwater of the Indian subcontinent during the late Neogene trend of sea‐level regression (Hamilton et al. 2001). Combined with the hypothesis that the tributaries started to shift from the Ganges nearly 5 MYA and completely connected to the Indus River around 0.3 MYA (Clift and Blusztajn 2005), this detected flourishment of the Indus River dolphin might be due to habitat expansion. There was an apparent reduction in *N*_e_ of the Indus River dolphin 40–20 kiloyears ago (KYA) (Figure 1B). This period is highly consistent with the upper Paleolithic, marked by the inconsistent monsoons and colder climate in the Indian context (Singhvi and Kale 2010; Khara et al. 2021), thus it might impact the habitat of the Indus River dolphin. This situation parallels that of the baiji, which also experienced a significant bottleneck approximately 10 KYA as a result of severe adverse environmental influences (Zhou et al. 2013). To advance understanding, it is necessary to obtain more high‐quality genomic data and employ various methods to estimate *N*_e_ of the Indus River dolphin across different time frames in the future.

It has been shown that populations with loss of genetic diversity and inbreeding can lead to sharp declines in adaptability, reproductive capacity, and disease resistance, thus heightening the risk of extinction (Lacy 1987). This observed genetic diversity of the Indus River dolphin is similar to the baiji (0.0121%) (Zhou et al. 2013), and is less than that of the pygmy right whale (*Caperea marginanta*) (0.11%) (Wolf et al. 2023), while it exceeds that of the minke whale ( *Balaenoptera acutorostrata* ) (0.00069%) (Yim et al. 2014), the sperm whale (0.0011%) (Warren et al. 2017), the fin whale ( *Balaenoptera physalus* ) (0.00142%), and the bottlenose dolphin (0.0014%). The heterozygosity of the Indus River dolphin is similar to that of the baiji, an extinct river dolphin over past decades, indicating its lower evolutionary potential. This highlights the need for increased attention to the survival status of the Indus River dolphin populations.

Further, the endangered populations facing the risk of anthropogenic impacts, such as habitat fragmentation, are usually threatened by population size reduction and inbreeding depression, leading to the lowered genetic diversity (Grueber et al. 2008). After 1885, the development of Indus Basin irrigation fragmented the overall habitat and population; additionally, hunting pressure declined the overall population to less than 200 individuals in the 1970s (Anderson 1878; Braulik et al. 2015). However, the subpopulation sizes increased rapidly over time. For example, during four decades from 1979 to 2017, the isolated subpopulation of the Taunsa‐Guddu barrage has expanded from 36 (Pilleri and Bhatt 1980) to more than 1000 individuals (WWF‐Pakistan 2017). The bottleneck effect of only a few generations is enough to lead to a loss of heterozygosity (Wolf et al. 2023). As the expected generation time of the Indus River dolphin is 8 years, about five generations have passed since 1979. Thus, the cumulative effect of historic and recent bottleneck events has a strong probability of the loss of heterozygosity due to inbreeding.

Cetaceans, including river dolphins, are a group of highly aquatic mammals that have evolved numerous remarkable adaptations (Würsig 1989; Springer et al. 2021). Thereinto, hair, a highly keratinized tissue in mammals, plays a crucial role in cetaceans by reducing friction and enhancing locomotion through significant evolutionary adaptations that lead to hair loss (Chen et al. 2013; Sun et al. 2017). We identified that the contracted gene families in the Indus River dolphin are significantly related to keratinization (GO:0031424), cornification (GO:0070268), and hair follicle morphogenesis (GO:0031069), supporting the evolution of the degenerated skin derivatives of the Indus River dolphin. Moreover, the degenerated olfactory system is another significant adaptation for fully aquatic life (De Vreese et al. 2023). The contracted gene families associated with olfactory receptor activity (GO:0004984) aligned with olfactory degeneration in the Indus River dolphin, as observed in another river dolphin, the baiji (Zhou et al. 2013).

Besides, river dolphins including *Platanista* have successfully adapted to and colonized the freshwater environment such as the Indus River system. Notably, in comparison to the marine ecosystem, the freshwater environment demonstrates a higher diversity and distinction of bacteria and other microorganisms (Auguet et al. 2010; Barberán and Casamayor 2010). In this study, we identified specific genes and gene families associated with immunity that have undergone positive selection and significant turnover, respectively (Figure 2C and Tables S7–S10). These findings indicated a specific immune response tailored to the freshwater environment of the Indus River dolphin.

## 5 Conclusion

The endangered status of the Indus River dolphin ( *P. minor* ) is attributed to the reduction in its distributional range, loss of habitat, and escalating severe anthropogenic pressures along the Indus River in Pakistan. The newly reported draft genome of the endangered species led to the identification of a peak in population size around 0.3–0.2 MYA, as indicated by the PSMC model. This finding implies a possible migration from the Ganges to the Indus River system at that time. As a consequence of climate change, the population experienced a gradual decline and encountered a bottleneck effect approximately 40–20 KYA. Additionally, our comparative genomic findings emphasize that the Indus River dolphin, residing in freshwater habitats, has evolved a highly adapted immune system. In conclusion, this study provides new insights into the molecular mechanisms involved in the freshwater adaptation, divergence, phylogeny, genetic diversity, and demographic history of the Indus River dolphin. These findings will contribute to conservation efforts for this species in the Indian subcontinent. In the future, advanced genome sequencing, comprehensive comparative, and population genomics studies are essential to elucidate the ecology, evolution, and adaptation of this species to freshwater. These studies will establish a robust scientific foundation for the conservation of this endangered species.

## Author Contributions

**Aamir Ibrahim:** conceptualization (equal), data curation (equal), writing – original draft (equal), writing – review and editing (equal). **Simin Chai:** conceptualization (equal), data curation (equal), visualization (lead), writing – original draft (equal), writing – review and editing (equal). **Cuijuan Zhong:** resources (equal), writing – review and editing (equal). **Kang Jieqiong:** investigation (equal), project administration (equal). **Ahsaan Ali:** investigation (equal), resources (equal). **Sajjad Hussain:** investigation (equal), resources (equal). **Hassan Ali:** investigation (equal), resources (equal). **Tanveer Hussain:** investigation (equal), resources (equal). **Umer Waqas:** investigation (equal), resources (equal). **Guang Yang:** conceptualization (lead), supervision (lead), writing – review and editing (equal).

## Conflicts of Interest

The authors declare no conflicts of interest.

## Supporting information


Figure S1‐S6

Table S1‐S13.

## Data Availability

The data is uploaded and available at the National Genomics Data Center (https://www.cncb.ac.cn/) with an accession number PRJCA027994.
