# Supplementary material for: Low Heterozygosity and Historical Bottleneck Effect Depicted From the Genome Assembly of the Indus River Dolphin ( Platanista minor )
Source: Ecol Evol. 2025 May 25;15(5):e71462. doi: 10.1002/ece3.71462 (PMC12103917; doi:10.1002/ece3.71462)
Supplement: Supplementary file 1 — Figure S1‐S6 [file ECE3-15-e71462-s002.docx]

**Low heterozygosity and** **historical bottleneck effect depicted from the genome assembly of the Indus River dolphin (*****Platanista minor*)**

Aamir Ibrahim^1,#^, Simin Chai^2,#^, Cuijuan Zhong^1^, Kang jieqiong^1^, Ahsaan Ali^1^, Sajjad Hussain^1,3^, Hassan Ali^3^, Tanveer Hussain^4^, Umer Waqas^5^, Guang Yang^1,2,*^

^1^Jiangsu Key Laboratory for Biodiversity and Biotechnology, College of Life Sciences, Nanjing Normal University, Nanjing, 210023, China

^2^Southern Marine Science and Engineering Guangdong Laboratory (Guangzhou), Guangzhou, Guangdong, 511458, China

^3^ Wildlife and Parks Department, Punjab, Pakistan

^4^Department of Biological Sciences, Virtual University of Pakistan, Islamabad

^5^Virtual University of Pakistan, Raiwand Road, Lahore, Pakistan

^#^Aamir Ibrahim and Simin Chai contributed equally to this work.

^*^Correspondence: Guang Yang, [gyang@njnu.edu.cn](mailto:gyang@njnu.edu.cn)


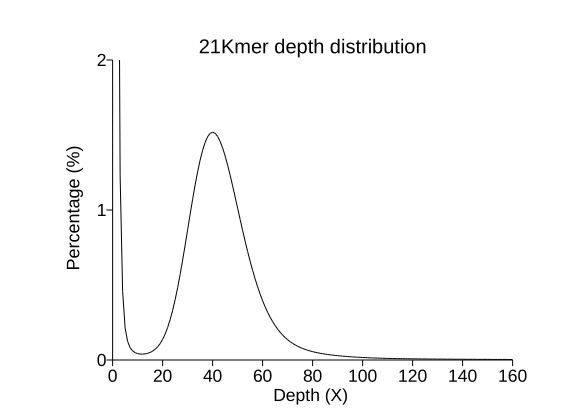
**Supplementary Figures**

Supplementary Figure 1. 21-mer frequency distribution of sequencing reads.


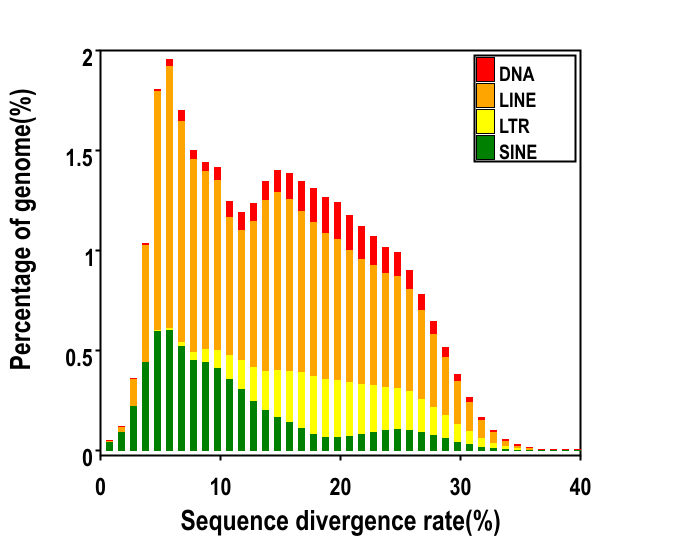


Supplementary Figure 2. Divergence histogram for repeated sequence classification.

**Supplementary Figure 3. The inferred phylogeny from concatenated alignments of the single-copy orthologues with bootstrap values.**


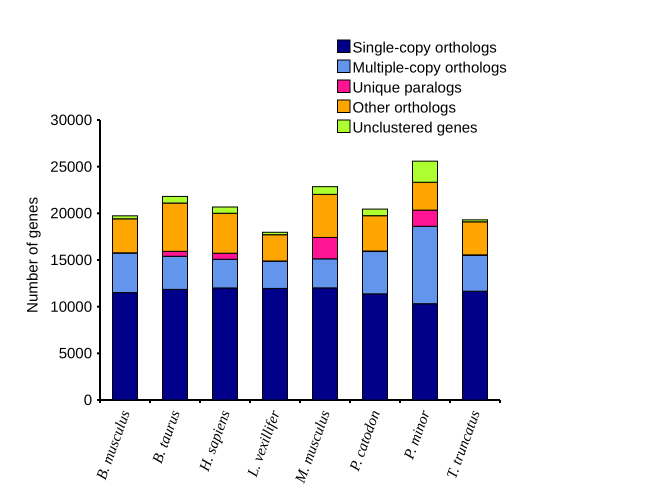


Supplementary Figure 4. Gene family column statistics result chart


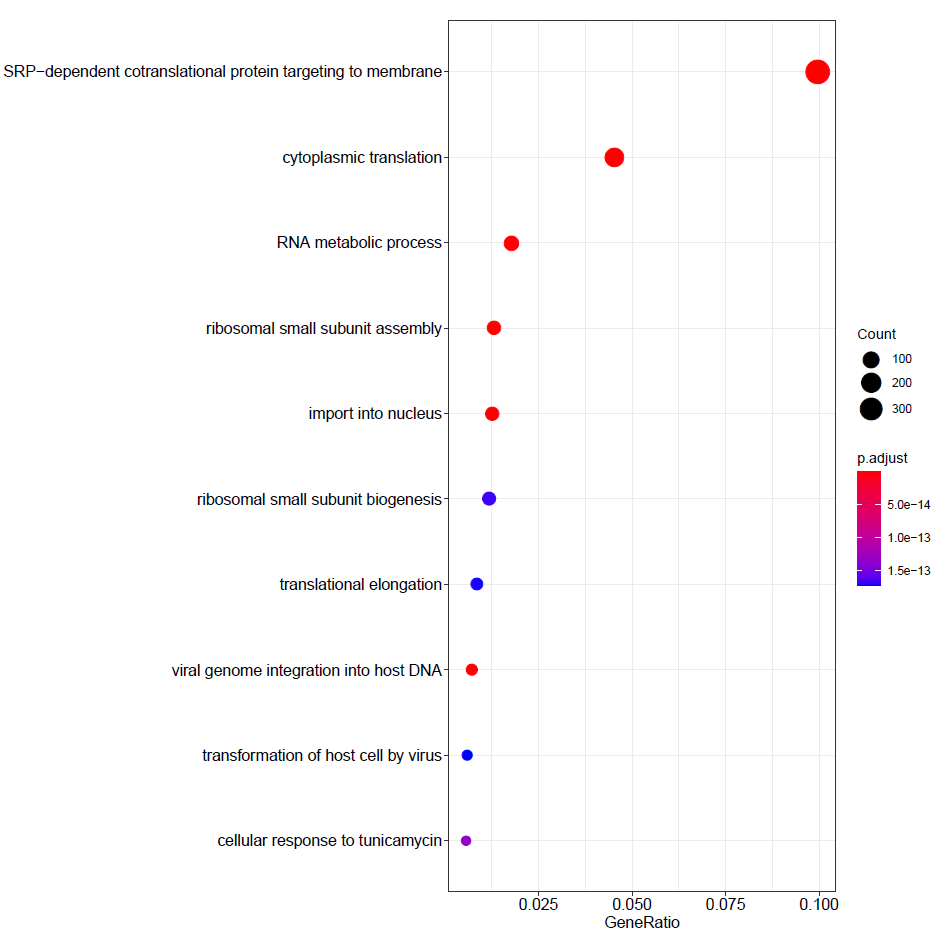


Supplementary Figure 5. Expanded gene GO terms (BP) enrichment bubble plot


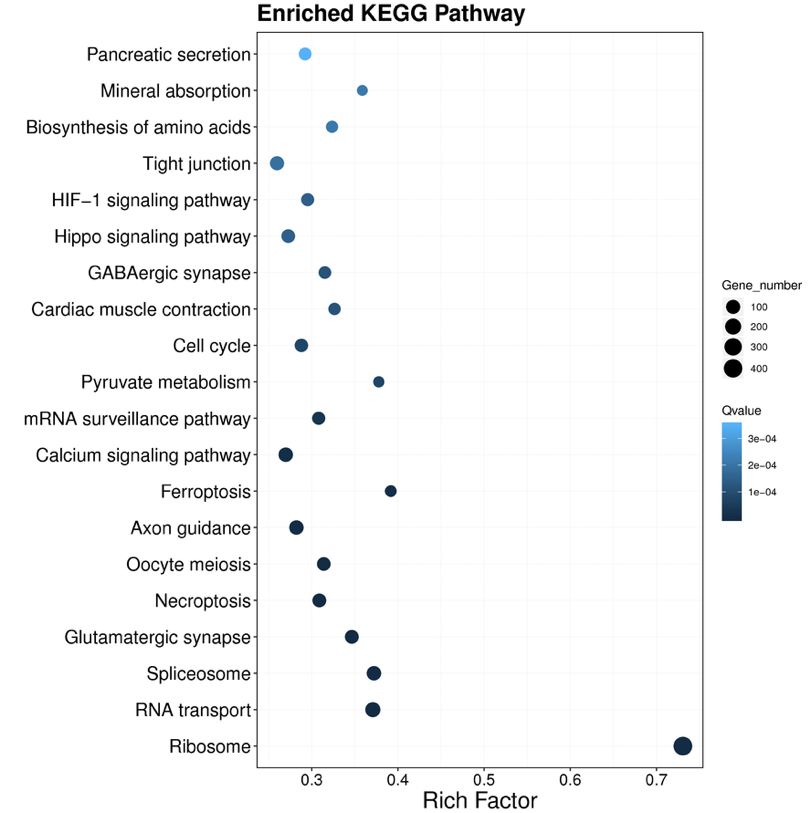


Supplementary Figure 6. Bubble chart of expansion gene KEGG enrichment results
