# Supplementary material for: Low Heterozygosity and Historical Bottleneck Effect Depicted From the Genome Assembly of the Indus River Dolphin ( Platanista minor )
Source: Ecol Evol. 2025 May 25;15(5):e71462. doi: 10.1002/ece3.71462 (PMC12103917; doi:10.1002/ece3.71462)
Supplement: Supplementary file 2 — Table S1‐S13. [file ECE3-15-e71462-s001.docx]

**Low heterozygosity and** **historical bottleneck effect depicted from the genome assembly of the Indus River dolphin (*****Platanista minor*)**

Aamir Ibrahim^1,#^, Simin Chai^2,#^, Cuijuan Zhong^1^, Kang jieqiong^1^, Ahsaan Ali^1^, Sajjad Hussain^1,3^, Hassan Ali^3^, Tanveer Hussain^4^, Umer Waqas^5^, Guang Yang^1,2,*^

^1^Jiangsu Key Laboratory for Biodiversity and Biotechnology, College of Life Sciences, Nanjing Normal University, Nanjing, 210023, China

^2^Southern Marine Science and Engineering Guangdong Laboratory (Guangzhou), Guangzhou, Guangdong, 511458, China

^3^ Wildlife and Parks Department, Punjab, Pakistan

^4^Department of Biological Sciences, Virtual University of Pakistan, Islamabad

^5^Virtual University of Pakistan, Raiwand Road, Lahore, Pakistan

^#^Aamir Ibrahim and Simin Chai contributed equally to this work.

^*^Correspondence: Guang Yang, [gyang@njnu.edu.cn](mailto:gyang@njnu.edu.cn)

**Supplementary Tables**

Supplementary Table 1. Final data after filtering and removing repetitive sequencing data

| **Lib ID** | **Insert Size**  **(bp)** | **Read Length**  **(bp)** | **Clean Data (Gb)** | **Q20(%)** |
| --- | --- | --- | --- | --- |
| FCHGL2CDSX7_L4 | 350 bp | 150b p | 265.31 | 97.59 |

Supplementary Table 2. 21-mer analysis data statistics

| **K** | **K-number** | **Peak** | **Genome size** | **Used base** | **Read length** | **Node number** |
| --- | --- | --- | --- | --- | --- | --- |
| 21 | 141,425,542,400 | 40 | 3,535,638,560 | 17,678,192,800 | 150 | 4,609,423,118 |

Supplementary Table 3. Statistics of Platanus assembly in the present study

| **Feature** | **Number of sequences** | **Length (bp)** |
| --- | --- | --- |
| N90 | 130 | 928,484 |
| N80 | 9,923 | 56,077 |
| N70 | 20,769 | 37,720 |
| N60 | 30,472 | 26,894 |
| N50 | 40,566 | 19,090 |
| N40 | 51,813 | 13,099 |
| N30 | 65,696 | 8,379 |
| N20 | 84,245 | 4,670 |
| N10 | 114,567 | 1,834 |
| Total | | **2,746,718,295** |

Supplementary Table 4. Statistical results of repeated sequence annotation

| **TE_class** | **TE_number** | **TE_length (bp)** | **TE_percentage** |
| --- | --- | --- | --- |
| **Retroelements** | 1,790,372 | 716,641,435 | 30.70% |
| **SINEs:** | 758,694 | 150,561,256 | 6.45% |
| **LINEs:** | 745,564 | 460,963,397 | 19.75% |
| **CRE/SLACS** | 262 | 15,047 | 0.00% |
| **L2/CR1/Rex** | 97,668 | 27,718,874 | 1.19% |
| **R1/LOA/Jockey** | 1,174 | 60,675 | 0.00% |
| **R2/R4/NeSL** | 669 | 53,229 | 0.00% |
| **RTE/Bov-B** | 1,547 | 136,618 | 0.01% |
| **L1/CIN4** | 633,894 | 432,217,821 | 18.51% |
| **LTR_elements:** | 286,114 | 105,116,782 | 4.50% |
| **BEL/Pao** | 1,777 | 82,587 | 0.00% |
| **Ty1/Copia** | 6,136 | 304,193 | 0.01% |
| **Gypsy/DIRS1** | 20,868 | 1,956,445 | 0.08% |
| **Retroviral** | 253,104 | 101,888,696 | 4.36% |
| **DNA_transposons** | 318,616 | 55,296,244 | 2.37% |
| **hobo-Activator** | 167,840 | 33,961,949 | 1.45% |
| **Tc1-IS630-Pogo** | 66,029 | 16,309,341 | 0.70% |
| **MULE-MuDR** | 6,909 | 378,598 | 0.02% |
| **PiggyBac** | 814 | 218,832 | 0.01% |
| **Tourist/Harbinger** | 3,958 | 239,130 | 0.01% |
| **Other** | 2,421 | 113,277 | 0.00% |
| **Rolling-circles** | 7,576 | 517,298 | 0.02% |
| **Unclassified:** | 111,727 | 32,108,285 | 1.38% |
| **Small_RNA:** | 600,162 | 127,111,973 | 5.44% |
| **Satellites:** | 8,058 | 2,053,885 | 0.09% |
| **Simple_repeats:** | 536,532 | 20,878,644 | 0.89% |
| **Low_complexity:** | 86,080 | 4,223,706 | 0.18% |
| **Total_TE:** | | 831,982,582 | 35.64% |

Supplementary Table 5. ncRNA statistical results

| **Type** | **Number** | **Total_length** | **Average length** |
| --- | --- | --- | --- |
| rRNA | 180 | 55962 | 310.9 |
| snRNA | 672 | 74414 | 110.74 |
| tRNA | 1063 | 78205 | 73.57 |
| miRNA | 474 | 37723 | 79.58 |

Supplementary Table 6. Statistical table of gene annotation Maker integration results

| **Gene_number** | **Average_gene_len.** | **Average_cds_len.** | **Average_exon_number** | **Average_exon_len** | **Average_intron_len** |
| --- | --- | --- | --- | --- | --- |
| 25,591 | 9,640.82 | 963.36 | 5.51 | 174.86 | 1,750.31 |

Supplementary Table 7. Gene family statistical results table

| **Name** | **single_copy orthologs** | **multi_copy orthologs** | **unique_copy orthologs** | **other_copy orthologs** | **Unclustered orthologs** |
| --- | --- | --- | --- | --- | --- |
| *B. musculus* | 11,506 | 4,246 | 15 | 3,645 | 321 |
| *B. taurus* | 11,850 | 3,547 | 532 | 5,177 | 706 |
| *H. sapiens* | 12,006 | 3,072 | 648 | 4,283 | 669 |
| *L. vexillifer* | 11,956 | 2,922 | 16 | 2,814 | 263 |
| *M.musculus* | 12,014 | 3,112 | 2,288 | 4,616 | 828 |
| *P. catodon* | 11,373 | 4,534 | 54 | 3,785 | 708 |
| *P. minor* | 10,315 | 8,294 | 1,735 | 2,985 | 2,262 |
| *T.truncatus* | 11,656 | 3,840 | 53 | 3,542 | 212 |

Supplementary Table 8. Expansion and contraction gene family

| **Branch** | **Expansions** | | **Contractions** | |
| --- | --- | --- | --- | --- |
|  | **Families** | **Genes** | **Families** | **Genes** |
| *B. musculus* | 732 | 886 | 1,774 | 1792 |
| *P. catodon* | 962 | 1,152 | 1,569 | 1,609 |
| *L. vexillifer* | 288 | 319 | 1,259 | 1,394 |
| *T. truncatus* | 522 | 752 | 437 | 443 |
| *P. minor* | 3,022 | 5,476 | 2,306 | 2,449 |
| *B. taurus* | 792 | 2,251 | 1,773 | 1,803 |
| *M. musculus* | 792 | 3,474 | 2,590 | 2,726 |
| *H. sapiens* | 559 | 1,481 | 2,611 | 2,758 |

Supplementary Table 9. GO (BP) Enrichment of the expanded gene families

| **ID** | **Description** | ***p*-value** | ***p-*adjust** |
| --- | --- | --- | --- |
| GO:0006614 | SRP-dependent cotranslational protein targeting to membrane | 3.67E-204 | 1.62E-200 |
| GO:0002181 | cytoplasmic translation | 1.02E-89 | 2.25E-86 |
| GO:0051170 | import into nucleus | 5.37E-25 | 7.89E-22 |
| GO:0000028 | ribosomal small subunit assembly | 3.63E-22 | 4.00E-19 |
| GO:0016070 | RNA metabolic process | 3.22E-21 | 2.84E-18 |
| GO:0044826 | viral genome integration into host DNA | 6.23E-21 | 4.58E-18 |
| GO:1904577 | cellular response to tunicamycin | 2.13E-16 | 1.34E-13 |
| GO:0042274 | ribosomal small subunit biogenesis | 3.03E-16 | 1.67E-13 |
| GO:0006414 | translational elongation | 3.51E-16 | 1.72E-13 |
| GO:0019087 | transformation of host cell by virus | 3.93E-16 | 1.73E-13 |

Supplementary Table 10. KEGG enrichment of the expanded gene families

| **#Pathway** | **P-value** | **Q-value** | **Pathway ID** | **Level 1** | **Level 2** |
| --- | --- | --- | --- | --- | --- |
| Ribosome | 4.68E-199 | 1.16E-196 | ko03010 | Genetic Information Processing | Translation |
| RNA transport | 5.95E-21 | 7.35E-19 | ko03013 | Genetic Information Processing | Translation |
| Spliceosome | 5.00E-17 | 4.12E-15 | ko03040 | Genetic Information Processing | Transcription |
| Glutamatergic synapse | 2.17E-11 | 1.34E-09 | ko04724 | Organismal Systems | Nervous system |
| Necroptosis | 1.48E-08 | 7.30E-07 | ko04217 | Cellular Processes | Cell growth and death |
| Oocyte meiosis | 2.41E-08 | 9.94E-07 | ko04114 | Cellular Processes | Cell growth and death |
| Axon guidance | 3.52E-08 | 1.24E-06 | ko04360 | Organismal Systems | Development |
| Ferroptosis | 3.06E-07 | 9.44E-06 | ko04216 | Cellular Processes | Cell growth and death |
| Calcium signaling pathway | 3.62E-07 | 9.93E-06 | ko04020 | Environmental Information Processing | Signal transduction |
| mRNA surveillance pathway | 1.18E-06 | 2.90E-05 | ko03015 | Genetic Information Processing | Translation |

Supplementary Table 11. Statistical results of genes subject to positive selection

| **Species** | ***P*-value= 0.05** | ***P*-value= 0** |
| --- | --- | --- |
| *P. minor* | 878 | 425 |

Supplementary Table 12. GO terms (BP) enrichment statistical table of genes subject to positive selection pressure

| **ID** | **Description** | **Gene Ratio** | **Bg Ratio** | ***P*-value** | ***Q-*value** |
| --- | --- | --- | --- | --- | --- |
| GO:1900016 | negative regulation of cytokine production involved in inflammatory response | 7/808 | 30/22844 | 6.74E-05 | 0.180772 |
| GO:0010656 | negative regulation of muscle cell apoptotic process | 5/808 | 17/22844 | 0.000237 | 0.180772 |
| GO:1903003 | positive regulation of protein de ubiquitination | 4/808 | 10/22844 | 0.000275 | 0.180772 |
| GO:0010954 | positive regulation of protein processing | 6/808 | 27/22844 | 0.000301 | 0.180772 |
| GO:1903799 | negative regulation of production of miRNAs involved in gene silencing by miRNA | 5/808 | 18/22844 | 0.000319 | 0.180772 |
| GO:0060055 | angiogenesis involved in wound healing | 6/808 | 30/22844 | 0.000551 | 0.247952 |
| GO:0090501 | RNA phosphodiester bond hydrolysis | 4/808 | 12/22844 | 0.000613 | 0.247952 |
| GO:0044828 | negative regulation by host of viral genome replication | 4/808 | 13/22844 | 0.00086 | 0.268687 |
| GO:2000320 | negative regulation of T-helper 17 cell differentiation | 4/808 | 13/22844 | 0.00086 | 0.268687 |
| GO:0006895 | Golgi to endosome transport | 8/808 | 58/22844 | 0.000948 | 0.268687 |
| GO:0009062 | fatty acid catabolic process | 5/808 | 23/22844 | 0.001082 | 0.278661 |

Supplementary Table 13. KEGG enrichment of positively selected genes

| **#Pathway** | ***P*-value** | ***Q*-value** | **Pathway ID** | **Level 1** | **Level 2** |
| --- | --- | --- | --- | --- | --- |
| Valine, leucine and isoleucine biosynthesis | 0.00140428 | 0.314559 | ko00290 | Metabolism | Amino acid metabolism |
| Steroid biosynthesis | 0.0050718 | 0.568041 | ko00100 | Metabolism | Lipid metabolism |
| Renin-angiotensin system | 0.00845218 | 0.631096 | ko04614 | Organismal Systems | Endocrine system |
| Fatty acid elongation | 0.04869607 | 1 | ko00062 | Metabolism | Lipid metabolism |
| Pantothenate and CoA biosynthesis | 0.09637838 | 1 | ko00770 | Metabolism | Metabolism of cofactors and vitamins |
| Lysosome | 0.1514236 | 1 | ko04142 | Cellular Processes | Transport and catabolism |
| Autophagy - animal | 0.1787568 | 1 | ko04140 | Cellular Processes | Transport and catabolism |
| Cytokine-cytokine receptor interaction | 0.185389 | 1 | ko04060 | Environmental Information Processing | Signaling molecules and interaction |
| ErbB signaling pathway | 0.2147004 | 1 | ko04012 | Environmental Information Processing | Signal transduction |
